# *Sphingobacterium spiritivorum* infection in a patient with end stage renal disease on haemodialysis

**DOI:** 10.1186/s12941-016-0141-5

**Published:** 2016-04-18

**Authors:** Amit Gupta, Julie Logan, Nada Elhag, Mike Almond

**Affiliations:** Southend University Hospital, Prittlewell Chase, Westcliff-on-Sea, SS0 0RY UK; Antimicrobial Resistance and Healthcare Associated Infections (AMRHAI) Reference Unit, Public Health England, London, NW9 5EQ UK

## Abstract

**Background:**

*Sphingobacterium spiritivorum* is a microorganism that is ubiquitously found in the environment. However, it is rarely isolated from human clinical specimens. There are few reports to date of *Sphingobacterium spiritivorum* causing disease in humans.

**Case report:**

We describe a case of *Sphingobacterium spiritivorum* infection in a patient on haemodialysis, which to our knowledge, has not been described before. Further testing revealed this strain was sensitive to multiple antimicrobials.

**Conclusion:**

Despite interrupted courses of several antibiotics, our patient clinically made a good recovery and continued to receive haemodialysis.

## Background

An 80 year old woman with end stage renal failure, secondary to type 2 diabetes mellitus, was established on long term unit based haemodialysis via a tunneled central venous dialysis catheter. She experienced symptoms of a generalised infection with no specific signs on examination to identify a potential source. Her past medical history included *treated* hypertension and calcified valvular heart disease.

Initial blood tests showed: haemoglobin 8.0 g/dL; white cell count 7.9 × 10^9^/L (neutrophils 5 × 10^9^/L); platelet 198 × 10^9^/L; C-reactive protein 58 ml/L. A set of blood cultures from the dialysis line and haemodialysis circuit both grew gram negative rods from the aerobic bottle only. The microorganism, as identified by the “bioMérieux API^®^ 20 NE ID kit”, was determined to be *Sphingobacterium spiritivorum* (with 98.7 % certainty).

The same organism grew from two subsequent blood cultures which were referred to the Public Health England AMRHAI reference laboratory (ARMRL) for confirmation of identification and antibiotic sensitivities. Matrix-assisted laser desorption/ionization time of flight (MALDI-TOF) mass spectrometry, using Microflex LT with Biotyper v3.1 database (Bruker Daltonics, Bremen, Germany), provided identification for both isolates, with log score values of 2.273 and 2.265 matching with *S. spiritovorum* type strain DSM 11722^T^. Partial (~1100 bp) 16S rRNA gene sequencing for both isolates were 100 % identical to each other and matched at 99.9 % to the *S. spiritivorum* type strain NCTC 11386^T^ (GenBank accession no KU759703) [1].

Sensitivity testing by ARMRL revealed no MBL activity. Further analyses showed that this strain was sensitive to co-trimoxazole, meropenem, ceftazidime, ciprofloxacin and trimethoprim. This strain was tested resistant to aminoglyclosides, amoxicillin and piperacillin–tazobactam (Table 1). Carbapenemase detection was also sought by polymerase chain reaction technique for the reference lab’s current catalogue of genes for class A (KPC, IMI, NMC, GES and SME), class B (IMP, VIM, GIM, NDM, KHM, TMB, SIM and SPM) and class D (OXA-48 like) carbapenemases.

**Table 1:** ARMRL antibiotic sensitivity testing for *Sphingobacterium spiritivorum*

Antibiotic	Minimum inhibitory concentration (mg/L)	Sensitive/inhibitory/resistant
Amikacin	>64	Resistant
Gentamicin	16	Resistant
Tobramycin	>32	Resistant
Aztreonam	>64	Resistant
Ceftazidime	8	Sensitive
Imipenem	8	Inhibitory
Meropenem	1	Sensitive
Piperacillin	32	Resistant
Piperacillin/tazobactam	32	Resistant
Co-trimoxazole	0.064	Sensitive
Colistin	>32	Resistant
Ciprofloxacin	1	Inhibitory
Minocycline	0.5	Undetermined

Minocycline activity could not be determined

The patient was initially commenced on trimethoprim for 5 days, followed by a 7 day course of meropenem. The patient did not tolerate either of these antibiotics due to severe side effects. A subsequent course of ciprofloxacin, was not well tolerated either for similar reasons.

Despite incomplete courses of antibiotics, the patient clinically made a good recovery. However, subsequent blood cultures a month later persistently grew *S. spiritivorum*. The source of infection was presumed to be the dialysis line as cultures from here were also positive, although this was not confirmed. After discussion with the patient, it was established that she would not consent for an elective dialysis line exchange. As a result of her well clinical status, plans were made to monitor her for signs of further infection and intervene with further antibiotics only if necessary.

No other patient on the dialysis unit was identified as having a similar infection.

## Discussion

After cardiovascular disease, infections are the second most common cause of morbidity and mortality in patients with end stage renal disease on haemodialysis. Other co-factors such as older age, diabetes, low serum albumin and dialysis through temporary venous catheters are all independent risk factors for infection and sepsis [2]. Peritoneal dialysis and haemodialysis through a fistula have lower rates of septicaemia than haemodialysis via intravenous catheters [3]. Interestingly, the incidence of pneumonia is also higher in patients on haemodialysis compared to those receiving peritoneal dialysis [4].

End stage renal disease imposes higher rates of infection due to a multitude of factors that may be associated with either one of impaired host immunity, enhanced bacterial virulence properties or risks inherent within haemodialysis [5]. In vitro and in vivo data demonstrates that uraemia impairs polymorphonuclear cell chemotaxis, phagocytosis and cytotoxic activities [6]. Uraemic retention solutes such as parathyroid hormone, polyamines, angiongenin and complement factor D may all impair neutrophil activity by causing inappropriate expression of cell adhesion molecules that result in a transient leukopenia. Degranulation and release of reactive oxygen species can also lead to “cell exhaustion,” impairing any anti-microbial capabilities [7].

Abundant iron stores or iron overload are also associated with impaired immune cell function. Parkinnen et al. showed that routine intravenous iron injections in haemodialysis patients may enhance bacterial virulence properties and contribute to bacterial spread [8].

The process of haemodialysis may expose patients to bacterial infections through the repeated breaching of the protective skin barrier during needle insertion, colonisation of indwelling dialysis catheters or contamination of the dialysate [5]. Catheter tips may become infected as bacteria grow along the tract within skin or improper handling of the catheter ports. Intraluminal infection and indirect haematogenous spread from another infection focus may also cause catheter infection [9]. Internal jugular lines carry a lower risk of bacteraemia compared to femoral vein lines [10]. Catheters made from polyvinyl chloride or polyethylene are perhaps more susceptible to bacterial adherence when compared to those catheters made out of polytetralfuoroethylene, silicone elastomer or polyurethane [11].

*Sphingobacterium*, first classified as a distinct species of bacteria in 1983, are characterised by the high sphingophospholipid content within their cell walls [12]. Sphingobacterium species are non-fermentative, non-motile, non-spore-forming aerobic gram-negative bacilli. They are also oxidase and catalase positive. The organism produces yellow colonies on blood agar plate [13].

Infections caused by the then named “*Flavobacterium*” species were first described in 1980 [14]. Infection and septicaemia caused by *Sphingobacterium* have since been well described in a variety of settings, including peritonitis, nectrotising fasciitis and respiratory tract infections [15]. Although 15 species of *Sphingobacterium* have been identified to date, literature describes *Sphingobacterium multivorum*/*spiritivorum* as the only species that have been isolated from human clinical specimens [16]. Results here show that *S. spiritivorum* can reliably be identified by MALDI-TOF mass spectrometry and 16S rRNA gene sequencing.

*Sphingobacterium* are usually found within soil, stagnant water and vegetation. They are described as opportunistic pathogens, however, they may cause infections in immunocompetent hosts. Four cases of *S. spiritivorum* associated infection have been described thus far. Two of these cases were of cellulitis, one of extrinsic allergic alveolitis, and the most recent case of septicaemia in a patient with acute myeloid leukaemia [15, 17–19]. Death only occurred in the patient with leukaemia, whereas the other cases resolved after antibiotic therapy. The source of infection was only determined in the patient with extrinsic allergic alveolitis, as the strain of bacteria isolated from patient sputum was isolated from a water reservoir within his steam iron [18].

*Sphingobacterium multivorum* septicaemia was originally described in a haemodialysis patient in 1984 [20]. There are, however, no cases of *S. spiritivorum* infections in the context of chronic kidney or end stage renal disease or dialysis. The source of haemodialysis catheter colonisation in this case was not determined. It is assumed that the patient was susceptible to opportunistic pathogens as a result of her comorbidities and her dialysis status.

The mainstay of treatment in previous cases has been antibiotic therapy with either combination cephalosporins with either penicillins or ciprofloxacin. *Sphingobacterium* are generally intrinsically resistant to aminoglycosides and polymyxin B [21]. Antibiotic sensitivity testing by multiple sources suggests Sphingobacterium are sensitive to quinolones and trimethoprim–sulfamethoxazole with *S. spiritivorum* having further sensitivities to carbapenems and some cephalosporins [15].

Data suggests that there are always inherent and unavoidable risks with implantable dialysis catheters, however, fully implanted devices such as Lifesite^®^ HD Access System have shown to cause fewer infective complications [10].

There has been some interest over the past decade in the prophylactic application of topical antibiotics at the dialysis catheter site. A meta-analysis covering 2445 patients revealed that when treated with topical mupirocin, there is a 78 and 66 % risk reduction in *Staphylococcus aureus* infection in patients on haemodialysis and peritoneal dialysis respectively [22]. Furthermore, a randomised controlled trial comparing the use of topical triple agent products such as polysporin, (bacitracin, gramicidin and polymyxin B), versus placebo showed a 60–65 % risk reduction in bacteraemia over a 6 month period [23]. With comparable results to that of mupirocin use, further studies are required to determine superiority and appropriate regimens.

Other potential infection preventative measures that are undergoing trials include catheter lock therapies, pneumonococcal and influenza vaccinations and pre-emptive recombinant human granulocyte colony stimulating factor.

## Conclusions

This report describes the first case of *S. spiritivorum* in a patient undergoing regular haemodialysis. Despite our patient not tolerating a wide range of antibiotics, she seemed to respond clinically and currently remains well on haemodialysis.
